# Dose linearity and uniformity of Siemens ONCOR impression plus linear accelerator designed for step-and-shoot intensity-modulated radiation therapy

**DOI:** 10.4103/0971-6203.35722

**Published:** 2007

**Authors:** Janhavi R. Bhangle, V. K. Sathiya Narayanan, Shrikant A. Deshpande

**Affiliations:** Department of Radiation Oncology, Ruby Hall Clinic, Pune, India

**Keywords:** Flatness, linearity, low MU, step-and-shoot intensity-modulated radiation therapy, symmetry

## Abstract

For step-and-shoot type delivery of intensity-modulated radiation therapy (IMRT), beam stability characteristics during the first few monitor units need to be investigated to ensure the planned dose delivery. This paper presents the study done for Siemens ONCOR impression plus linear accelerator before commissioning it for IMRT treatment. The beam stability for 6 and 15 MV in terms of dose monitor linearity, monitor unit stability and beam uniformity is investigated in this work. Monitor unit linearity is studied using FC65G chamber for the range 1-100 MU. The dose per MU is found to be linear for small monitor units down to 1 MU for both 6 and 15 MV beams. The monitor unit linearity is also studied with portal imaging device for the range 1-20 MU for 6 MV beam. The pixel values are within ±1σ confidence level up to 2 MU; for 1 MU, the values are within ±2σ confidence level. The flatness and symmetry analysis is done for both energies in the range of 1-10 MU with Kodak diagnostic films. The flatness and symmetry are found to be within ±3% up to 2 MU for 6 MV and up to 3 MU for 15 MV.

Intensity-modulated radiation therapy (IMRT) is becoming the commonly chosen treatment modality because of its dose conformity to the concave tumor volumes, sparing surrounding normal organs. Siemens ONCOR Impression Plus linear accelerator with photon beam energies of 6 and 15 MV equipped with double-focused multileaf collimator (MLC) has been installed in our clinic. These accelerators are designed to deliver intensity-modulated fields using step-and-shoot technique. In this type of IMRT delivery, multiple segments are the result of the optimization process to conform the dose to the target volume. These segments are delivered in a stack arrangement. Each beam segment is delivered separately with the radiation beam turned off (paused)[1] when the MLC moves to take a shape of the next segment. The composite of dose increment delivered to each segment delivers the planned intensity modulation. The accuracy of intensity modulation depends on the capabilities and limitations of the linear accelerator equipped with the multi-leaf collimator to deliver low monitor unit (MU) segments. For complex tumor-normal tissue configuration, IMRT plan can involve large numbers of field segments, leading to many very small fields and low doses per segment.

The operation of the linear accelerator delivering low monitor unit segments needs to be investigated for every linear accelerator prior to commissioning the linear accelerator for IMRT.[2] Many authors have studied the performances of different linear accelerators in low-MU region using different methods.[1,3–6] Dose linearity, uniformity and monitor unit stability in low-MU region for Siemens ONCOR Impression Plus linear accelerator for both 6 and 15 MV photon energies were investigated in this work.

## Materials and Methods

Dose monitor linearity and monitor unit stability at small monitor units for 6 and 15 MV were examined using a FC65G ionization chamber connected to DOSE1 electrometer. The measurements were done at 100 cm SSD with the chamber at d_max_ in the SP34 slab phantom.[1,4] The collimator settings were 10 cm × 10 cm. The measurements were done for 6 MV photon beam with dose rate of 300 MU/min and for 15 MV with dose rate of 500 MU/min. For low MU linearity, ionization readings were recorded for 100, 50, 20, 15, 10, 5, 3, 2 and 1 MU. These readings were normalized to 2 MU and to 100 MU reading, and the corresponding percentage variation for all other MUs was calculated.[3] The monitor unit linearity for 6 MV was also studied using OPTIVUE AG9 flat panel with 1024 × 1024 pixels. For the field size of 10 cm × 10 cm, portal images were obtained for 20, 15, 10, 5, 3, 2 and 1 MU with the flat panel at 145 cm. The statistical analysis of the data is presented.

Monitor unit stability was studied with the same setup as described above. For the collimator setting of 10 cm × 10 cm, the ionization was recorded for 10 MU. It was compared with the cumulative ionization of 5 MU measured two times; 2 MU measured five times, 1 MU measured ten times.[4] Similarly, ionization recorded for 15 MU was compared with the cumulative ionization of 5 MU measured three times; 3 MU measured five times, 1 MU measured fifteen times.

Film dosimetry was used to study dose uniformity for low monitor units.[5] Beam flatness and symmetry for 6 and 15 MV were evaluated with Kodak diagnostic film. For SFD 100 cm with collimator setting of 10 cm × 10 cm, exposures of 10, 5, 3, 2 and 1 MU were given. Vidar VXR16 Dosimetry Pro scanner, along with Omni-Pro IMRT software, was used to analyze these films for flatness and symmetry.[7]

## Results and Discussion

**Figure 1:**
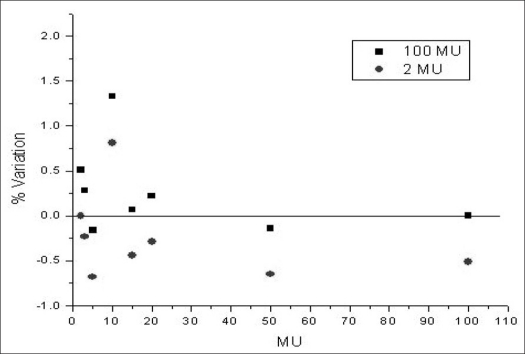
6 MV ionization readings normalized to 100 MU and 2 MU

For low-MU linearity, ionization readings were recorded in the range of 1-100 MU. Figure 1 shows the percentage variation when these readings were normalized to 2 MU reading and to 100 MU reading. The percentage variation in the relative ionizations is within 2% for low monitor units such as 1 MU.

**Figure 2:**
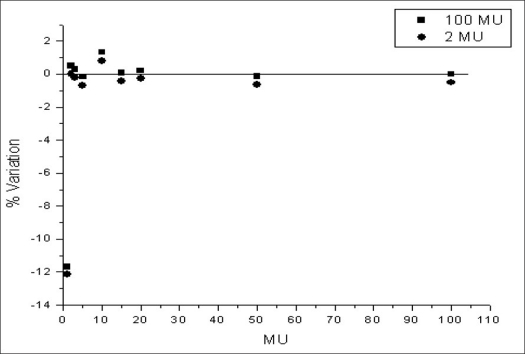
15 MV ionization readings normalized to 100 MU and 2 MU

Similar study done for 15 MV is shown in Figure 2. For 15 MV beam, the percentage variation in the relative ionization is within 2% up to 2 MU. For 1 MU the variation is high.

**Figure 3:**
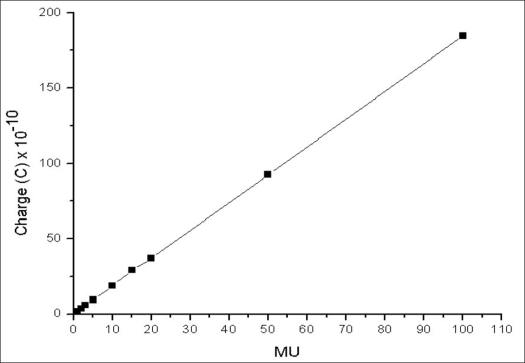
Monitor unit linearity for 6 MV

**Figure 4:**
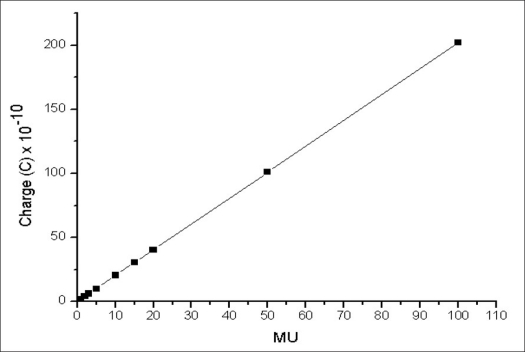
Monitor unit linearity for 15 MV

Figures 3 and 4 show linearity of charge measured as a function of monitor unit for 6 MV and 15 MV beam respectively. For both the energies, the charge is found to be linear down to 1 MU.

**Figure 5:**
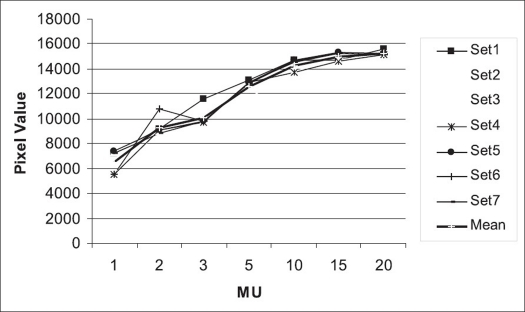
MU linearity in terms of pixel values for 6 MV

Figure 5 represents the change in the pixel values obtained from the portal imaging device as a function of monitor units in the range of 1-20 MU. It is observed that the pixel values are not exactly linear with monitor units. This can be because of the response of the flat panel. The flat panel gets saturated above 15 MU. The deviation from the mean curve is calculated for every data set. It is observed that the pixel values for 2-20 MU are within ±1σ confidence level. Only for 1 MU, the pixel values are within ±2σ confidence level.

Figures 6A and 6B show monitor unit stability for 6 MV. The variation in the cumulative ionization recorded for 5 MU, measured two times, is −1.0%. The variation in the cumulative ionization recorded for 2 MU, measured five times; and that for 1 MU, measured ten times, from the ionization recorded for 10 MU is within 1.0%. Similarly, for 15 MU the variation in the cumulative ionization recorded for 5 MU, measured three times, is −1.45%; while that measured for 3 MU five times and 1 MU fifteen times is 0.31 and 1.01% respectively.

**Figure 6A:**
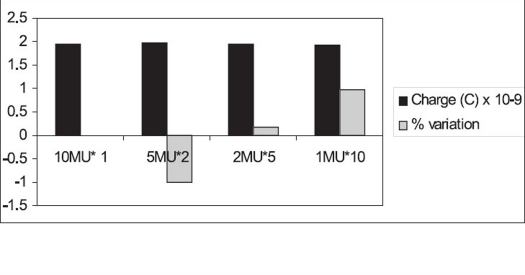
Monitor unit stability of 10 MU for 6 MV beam

**Figure 6B:**
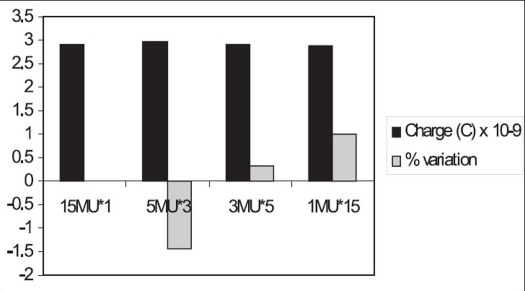
Monitor unit stability of 15 MU for 6 MV beam

For 15 MV [Figure 7A], the variation in the cumulative ionization recorded for 5 MU, measured two times, from the ionization recorded for 10 MU is 1.06%; for 2 MU, measured five times, the variation is 0.45%. But for the cumulative ionization recorded for 1 MU ten times, the variation is found to be 16%.

**Figure 7A:**
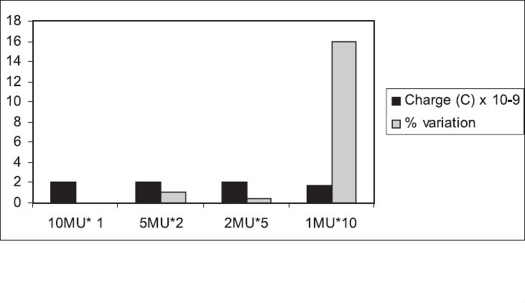
Monitor unit stability of 10 MU for 15 MV beam

For 15 MU, as shown in Figure 7B, the variation for 5 MU, measured three times; and 3 MU, measured five times, from the ionization recorded for 15 MU is 0.24 and −0.21% respectively. The variation in the cumulative ionization for 1 MU, measured fifteen times, from that of 15 MU is found to be 13.29%. The time required to deliver 1 MU of 15 MV at the dose rate of 500 MU/min is ∼ 120 msec, and that required for 1 MU of 6 MV at the dose rate of 300 MU/min is ∼ 200 msec. It is interesting to note that the ramp-up time specified by the manufacturer is 250 msec.[8] This could be one of the reasons for the high variation in the cumulative ionization of 1 MU, measured ten and fifteen times, for 6 and 15 MV.

**Figure 7B:**
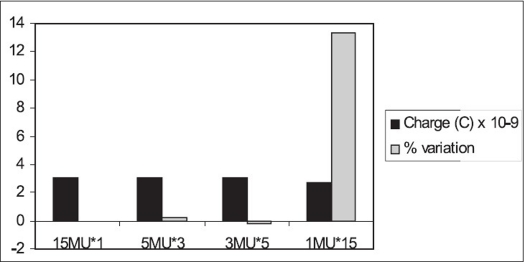
Monitor unit stability of 15 MU for 15 MV beam

**Figure 8:**
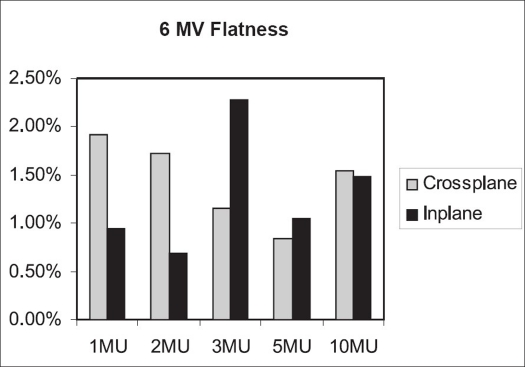
Inplane and crossplane flatness for 6 MV beam

**Figure 9:**
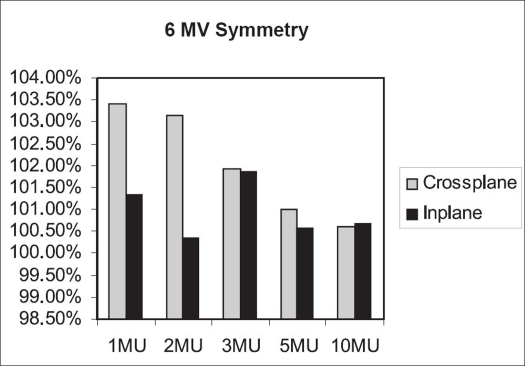
Inplane and crossplane symmetry for 6 MV beam

Flatness symmetry study was done with Kodak diagnostic films. Flatness was calculated using ‘variation over mean’ (80%) method. The symmetry value was obtained with ‘point difference quotient’ method.[5] For 6 MV beam [Figure 8], flatness in both planes is within 3%. The crossplane symmetry for 1 MU of 6 MV is 3.41%; and for 2 MU, 3.16%. For 3, 5 and 10 MU, the crossplane symmetry is within 2% for 6 MV; while the inplane symmetry values are within 2% for 1–10 MU [Figure 9].

**Figure 10:**
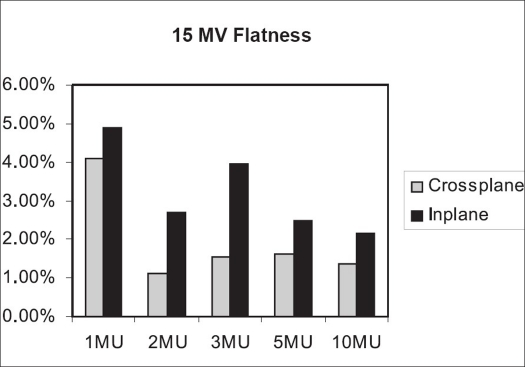
Inplane and crossplane flatness for 15 MV beam

For 15 MV beam, inplane and crossplane flatness for 1 MU is 4.11 and 4.88% respectively. For 2, 3 5 and 10 MU, the crossplane flatness values are within 2%. The inplane flatness is within 3% for 2, 5 and 10 MU; while it is 3.97% for 3 MU [Figure 10].

**Figure 11:**
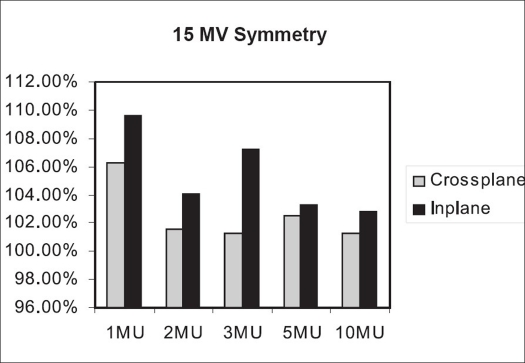
Inplane and crossplane symmetry for 15 MV beam

The symmetry analysis for 15 MV [Figure 11] shows that for 1 MU of 15 MV beam, the crossplane and inplane symmetry are 6.24 and 9.59% respectively. For 2, 3, 5 and 10 MU, the crossplane symmetry values are within 3%. The inplane symmetry for 2 MU is 4%; for 3 MU, 7%; for 5 MU, 3.3%; and for 10 MU, it is 2.84%.

## Conclusion

For 6 MV beam, the stability of the beam at low MU in terms of cGy/MU is within ±2%, assuming all other factors are proportional while converting the charge to cGy. The stability in terms of flatness and symmetry is also acceptable and is within ±3% up to 2 MU. The stability of 15 MV beam in terms of flatness and symmetry is within ±3% above 3 MU.

However, it is suggested that segments with monitor units less than 5 MU should be avoided. The inverse planning system used at our center takes care of prevention of small MU segments such as 5 MU from the treatment plan. So for the range of monitor units used for patient treatment, the Siemens ONCOR Impression Plus linear accelerator has been delivering stable beams for step-and-shoot IMRT treatments.
